# Fragility Fractures and Parkinsonism: Relationship of Fractures with Demography, Severity and Predictors of Adverse Outcomes

**DOI:** 10.3390/geriatrics2020017

**Published:** 2017-05-24

**Authors:** Shridhar Aithal, Ruford Sequeira, Chris Edwards, Inderpal Singh

**Affiliations:** 1 Department of Geriatric Medicine, Ysbyty Ystrad Fawr, Aneurin Bevan University Health Board, Wales CF82 7EP, UK; Shridhar.Aithal@wales.nhs.uk; 2 Geriatric Medicine, Aneurin Bevan University Health Board, Wales CF82 7EP, UK; Ruford.Sequeira3@wales.nhs.uk; 3 Consultant Clinical Scientist, Royal Gwent Hospital, Aneurin Bevan University Health Board, Newport NP20 2UB, UK; Chris.Edwards3@wales.nhs.uk

**Keywords:** fragility fracture, osteoporosis, Parkinson’s disease, disease severity, disease duration

## Abstract

**Background:** The risk of falls is higher in patients with Parkinsonism (PwP) as compared to other older adults, leading to adverse outcomes including fragility fractures. Osteoporosis is under-recognised and the current prevalence of fragility fractures is not well-studied. The objectives of this study are to determine the prevalence of fragility fractures in PwP, to measure the relationship of fractures with demography, severity and to measure predictors of adverse outcomes in this population. **Method/Description:** This was a retrospective observational cohort study based on the analysis of existing data for all the patients attending Caerphilly Movement Disorder Clinic. Information on demographics, the severity of Parkinsonism and fragility fractures was extracted electronically from the clinical workstation, clinic letters and coding from January 2015 to October 2016. **Results:** 397 people (mean age = 77.1 ± 9.4, 46% females) were studied. Of these, 77% (306/397) had Parkinsonism and 80% (244/306) had idiopathic Parkinson’s disease (PD). The mean Hoehn & Yahr Score at the time assessment was 3.09 ± 1.16. Additionally, 23.5% (72/306) were deemed to have osteoporosis based on the radiological evidence of fragility fractures. The PwP who sustained fractures were comparatively older (80.4 ± 12.1) and 70% (50/72) were females. The most common site for fractures was vertebral (47.2%; 34/72). Also, 22.2% of the sample (16/72) had suffered a fragility fracture before the diagnosis of PD. However, a majority (77.8%; 56/72) had sustained a fracture during the course of PD with a mean lapse of 6 years (range = 0–13 years) from initial diagnosis. Only 40% (29/72) of patients were prescribed osteoporosis drugs as per guidelines. There is a significant correlation of advancing age, severity and duration of PD with fragility fractures. The single best predictor of mortality is severity of PD, followed by age and fractures. **Conclusions:** There is a high prevalence of fragility fractures in patients attending movement disorder clinics, although 60% do not receive evidence-based medical treatment for the underlying osteoporosis. The prevalence of fragility fractures is positively correlated with the duration and severity of PD. We acknowledge the relatively small sample size as the study’s limitation.

## 1. Introduction

Osteoporosis and Parkinsonism are two long-term conditions which affect a considerable percentage of older people. Most patients with Parkinsonism (PwP) experience falls [1], which increase the risk of admission of PwP to hospital and nursing homes [2]. The risk of falls is more than double in patients with Parkinson’s disease (PD) as compared to those without PD [3].

Fragility fractures, particularly of the hip, are associated with poor outcome, and survival rates decline following hip fracture. Nearly one tenth will die within one month of hip fracture, and a third will die during the next one year [4]. Fewer than half will regain their pre-admission level of independence or return to their own home [4,5].

Osteoporosis is closely associated with PD [6,7,8,9,10], and has the strongest associations with fragility fractures above other long-term medical conditions [11]. Patients with PD have a significantly greater rate of age-adjusted mean annual total hip bone loss as compared to non-PD patients. PD patients also have higher rates of non-vertebral fractures [12] and mortality [7]. Overall, here is a high incidence of hip fracture in patients with PD [8,13,14,15,16].

PD not only increases the risk of falls, but also is associated with poor clinical outcomes including fragility fractures [11,17], hospital admission, and institutionalisation [6]. Fragility fractures are often under-diagnosed in PD, and current prevalence of fragility fractures in PwP warrants further study [8,17]. The objectives of this study are to determine the prevalence of fragility fractures in PwP, to measure the relationship of fractures with demography and severity and also to measure predictors of adverse outcomes in this population attending the geographically defined Caerphilly Movement Disorder (MD) Clinic.

## 2. Methods

### 2.1. Study Design

This was a retrospective cohort study. The study was based on an analysis of the existing data for all the patients attending the geographically defined Caerphilly Movement Disorder Clinic.

### 2.2. Setting

This was a preliminary study from one hospital (Ysbyty Ystrad Fawr) within Aneurin Bevan University Health Board (ABUHB), which has two other sites providing similar MD services.

### 2.3. Data and Statistical Analysis

The existing data for all the patients with Parkinsonism (PwP) attending the Caerphilly Movement Disorder Clinic were included in the analysis. This clinic serves a clearly defined and relatively static population of 180,200 within a specific geographic region: Caerphilly County Borough, Wales, UK. 

Clinical data including demography, co-morbidities, the severity of PD, disease duration and mortality were extracted from the existing Health Board electronic records and clinic letters from March 2015 to February 2016. Information on fragility fractures was retrieved from the existing X-ray records. The duration of PD was measured from the date of diagnoses to 20 October 2016. Mortality data was reviewed for at least six months, up to 20 October 2016. 

The disease severity was assessed using Hoehn and Yahr (H & Y) Score [18]. The score was measured based on the last MD clinic assessment. Stage 0: no signs of PD; stage I: unilateral signs with no impairments for activities of daily living; stage II: signs predominant unilateral signs with some impairment; stage III: bilateral signs with some postural instability; stage IV: severely disabled but can walk; and stage V: Wheelchair or bed-bound.

The description of the study cohort was completed and data were presented as means ± standard deviation. The level of statistical significance at which the null hypothesis was rejected was chosen as 0.05. All statistics were conducted using STATISTICA StatSoft data analysis software system, version 9.1 (StatSoft, Inc., 2010, Tulsa, OK, USA).

Sub-analysis was done to measure the relationship of age, duration of PD and severity of PD on fragility fractures using analysis of variance (ANOVA). 

Sub-analysis was also explored to measure the predictive effects of age, gender, PD severity (as measured by the H & Y score) and history of fracture on mortality using a Binomial Linear Model with logit link function.

Ethical approval was not required for this service evaluation as this work does not constitute a research study according to the Health Research Authority decision tool; however, all questions and forms required to carry out the study were sent to the ABUHB Research and Development (R & D) Department, to assess risks to patient identification and the health board. The study was approved by the R & D as a service evaluation as patients were not directly interviewed and no identifiable patient data was recorded. 

**Results:** The study population included a cohort of 397 people (mean age = 77.1 ± 9.4 years, 46% females) under follow-up in the MD clinic. Of these, 77% (306/397) had Parkinsonism (mean age = 77.7 ± 9.67 years, 41.8% females). The mean age of females (41.8%, *n* = 128/306) was 79.03 ± 10.65 years, which was significantly higher than that of males (58.2%, *n* = 178/306, mean age = 76.77 ± 8.78 years). In addition, 80% (244/306) had idiopathic PD with symptom onset between 1998 and 2016 and were followed until the date October 20, 2016, or death. Eleven percent had been diagnosed for more than 10 years. The mean Charlson’s Comorbidity Index was 1.5 ± 1.7 at the time of initial diagnosis of Parkinsonism, and the majority were on polypharmacy. The mean H & Y Score at the time of assessment was 3.09 ± 1.16.

The prevalence of fragility fractures was 23.5% (72/306) and the mean age on suffering fracture was 80.4 ± 12.1 years. Of this sample, 70% (*n* = 50/72) of these were females. In this cohort of community-dwelling people with PD, vertebral fractures were the most common type of fragility fractures, accounting for 47.2% (34/72). The incidence of non-vertebral fractures was as follows: hip 27.8% (20/72); wrist 18.1% (13/72); pelvis 4.2% (3/72) and humerus 2.8% (2/72). Only 40% (29/72) were on appropriate treatment as per guidelines. In addition, one patient had confirmed osteoporosis by Dual-energy X-ray absorptiometry (DXA) scan and 8% of patients (24/306) had osteopenia reported by X-rays. 

In the study population, 22.2% of subjects (*n* = 16/72) had suffered a fracture before the diagnosis of PD, but a majority (77.8%; *n* = 56/72) had sustained fragility fracture during the course of PD with a mean lapse of 6 years (range = 0–13 years) from the initial diagnosis. 

**Figure 1:**
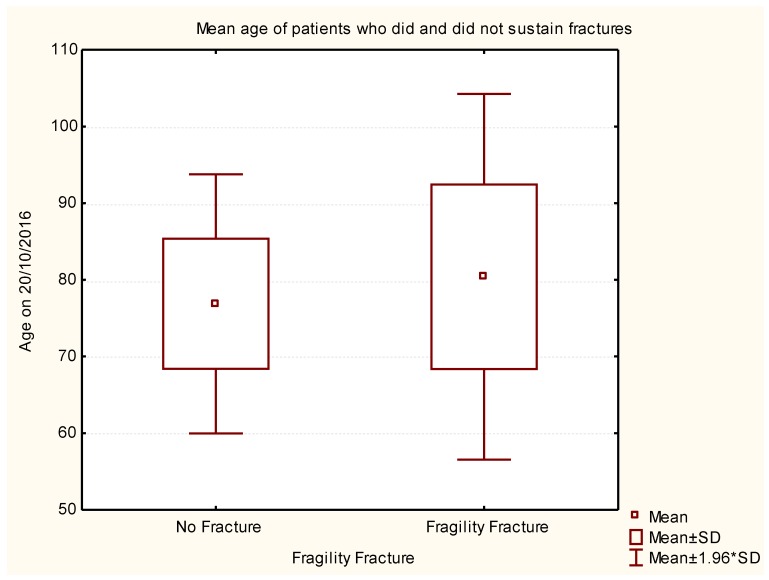
Box-and-whisker plot representing statistical data to correlate age with and without fragility fracture.

The mean age of the PwP who sustained fragility fracture was 80.40 ± 12.16 years, which was significantly higher as compared to those without fragility fractures (mean age = 76.87 ± 8.62 years, *p* < 0.006) (Figure 1). 

The history of previous fragility fracture in PwP was associated with gender and a significantly greater proportion of females had sustained previous fragility fracture than males (males 22/178, 12.4%; females 50/128, 39.1%, a highly significant difference between proportions test, *p* < 0.001).

**Figure 2:**
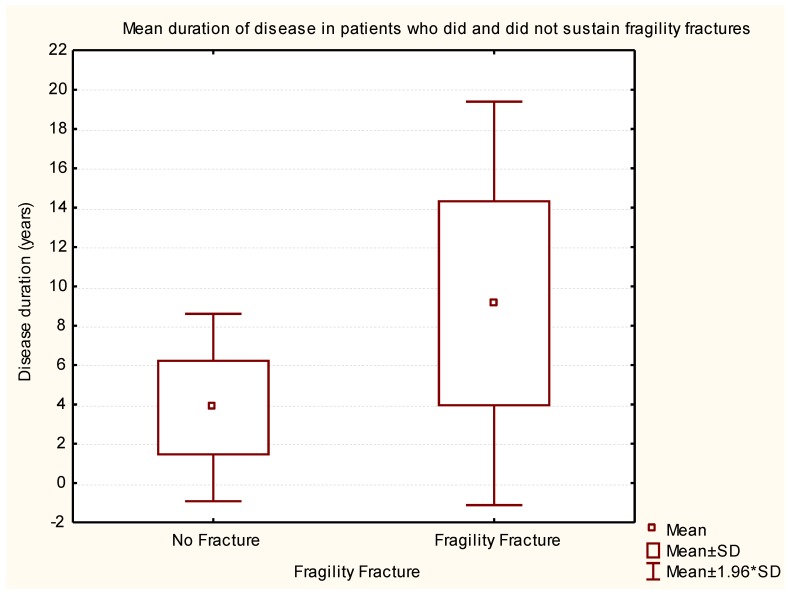
Box-and-whisker plot representing statistical data to correlate the duration of Parkinsonism with and without fragility fracture.

The mean duration of PD for those with and without fragility fractures was 9.14 ± 5.23 and 3.84 ± 2.43 years, respectively. There was a statistically significant correlation between a longer duration of PD and fragility fractures (*p* < 0.0001) (Figure 2).

**Figure 3:**
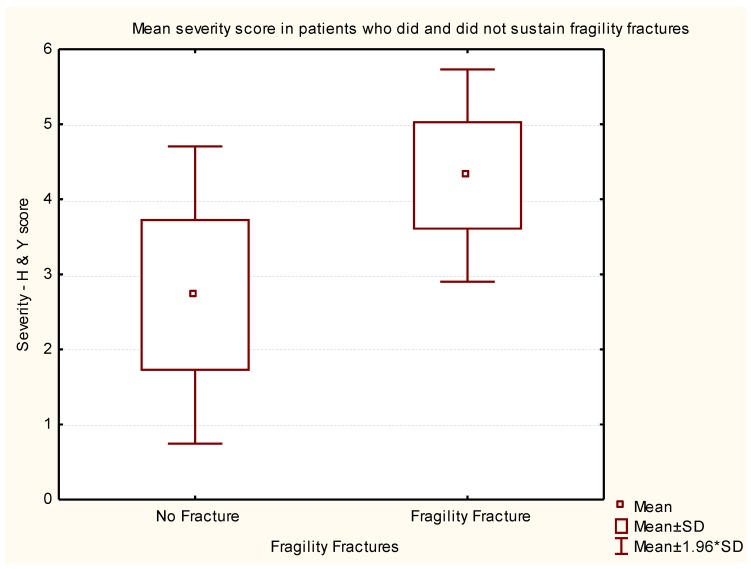
Box-and-whisker plot representing statistical data to correlate the severity of Parkinson’s disease with and without fragility fracture.

The severity of PD was measured by the H & Y Score at the time of the study. ANOVA test was used to complete the sub-analysis to measure the impact of the current severity of PD. PwP who sustained fragility fractures had an average H & Y Score of 4.3 ± 0.7 (*n* = 66), and those without fragility fractures had an average H & Y Score of 2.7 ± 1.0, (*n* = 219). The severity of PD was significantly related to the history of previous fragility fractures. (*p* < 0.001, ANOVA test, *p* ≤ 0.0001 Tukey’s Honestly Significant Difference (HSD) test (Figure 3).

Overall, 14% of patients (*n* = 43/306) had died by October 20, 2016. The mean age at death was 83.8 ± 7.0 years and the average time from the onset of symptoms to death was 7.1 ± 4.3 years. The likelihood or risk of death was higher for those patients who were older when their Parkinson’s disease was diagnosed.

Furthermore, 60.5% of patients (*n* = 26/43) who died did not have a fragility fracture. Patients who had fragility fractures and died accounted for 39.5% (*n* = 17/43), whereas those who had fragility fractures but remained alive accounted for 21% (*n* = 55/262) of the study sample. Sub-analysis of the difference between proportions test was not significant (*p* = 0.126). 

Sub-analysis was also completed to examine which of our observations of age, severity and duration of disease were predictors of the fracture outcome. This was a binary outcome (yes or no) rather than a continuous variable, so correlation with our predictors was not appropriate. However, we explored the predictive effect of our continuous predictors on the fracture outcome employing a generalised linear model using a binomial distribution with a logit link function. This model shows that the best single predictors of a fragility fracture are disease severity and disease duration. The strongest prediction uses all three predictors, but the age of the patient does not add much predictive power. All the models, showing the effects of using single or multiple predictor variables including age, duration of disease and H & Y score are summarized in the Table 1.

In addition, sub-analysis was conducted to understand the predictors of mortality. The single best predictor of mortality was PD severity, followed by age and then fracture history. All of these were considered statistically significant (H & Y *p* < 0.001; Age *p* < 0.001; Fracture *p* = 0.006). Gender was not a significant predictor of mortality in these patients.

## 3. Discussion

Parkinson’s disease (PD) is a progressive neurodegenerative disorder characterised by bradykinesia, rigidity, tremor and postural instability. The prevalence of PD is increasing and older people are more commonly affected. The prevalence of PD is nearly 1% among people aged 65 to 69 years, rising up to 3% among older people over 80 years [19]. We expect to see a higher number of PD patients due to ageing populations and improved survival in view of advances in diagnosis and management of PD. There is an exponential increase in the falls risk with age, from 35% in older adults above 65 years of age, to 45% in older people over the age of 80 years [20]. 

Disorders of gait and balance are the most common neurological diagnoses associated with falls [21]. The risk of falls is particularly associated with PD (62%), which is higher than other neurological disorders including polyneuropathy (48%), spinal disorders (41%) or stroke (22%) [22]. The most common risk factors leading to falls in PwP include older age, bradykinesia, rigidity, gait disturbances, postural instability, longer duration of the disease and severity of PD [3,23]. Atypical Parkinsonism and associated dementia increases the risk proportionately [3,24].

The prevalence of osteoporosis/osteopenia is high in PwP [25,26]. This study supports previously reported findings that PD is an important associated risk factor for fragility fractures. The most common reported fracture in PD patients in literature is of the femur [8], but we observed that vertebral fractures (11%) were the most common fractures among our study population. The overall prevalence of fragility fractures in this study was 23.5% and 6.5% PwP had a hip fracture. Hip fracture has been reported as high as 10% in patients with PD and, in comparison, only 4.1% of patients without PD sustained a fracture during the eight year follow-up period [25,26,27]. The observed increased risk of fragility fractures in PD has important clinical implications in terms of providing comprehensive, person-centred care.

The findings of this study support the correlation of advancing age, female gender, and duration or severity of the disease as measured by H & Y Scoring with fragility fracture. However, the best single predictors of a fragility fracture are disease severity and disease duration. Age was a weak predictor of adverse outcome, and the age of the patient did not add much predictive power when added to other variables. Chronological age had not shown any significant association with adverse outcome in a study examining very frail patients admitted to hospital [28]. Previously, similar correlations of older age, female gender, duration and severity of the disease have been related to more severe osteoporosis in one study [26]. Although, in other studies, lower Bone Mineral Density (BMD) has been related only to the severity of the PD but was not associated with the longer duration of the disease [25]. Yet, another study reported no correlation between the severity of PD and BMD, but lower BMD was related to the duration of disease [14]. 

There are other factors in addition to advanced disease and longer duration of the disease, such as low body mass index (BMI), vitamin D deficiency, limited exposure to sunlight and secondary hyperparathyroidism, all of which could results in low BMD in PwP. These factors need further study and correlation with the prevalence of fragility fractures in PwP. 

Our study has numerous strengths. Fragility fracture data was collected from the existing electronic health board data and all the X-rays reports were reviewed for each patient included in the study. Only a few fractures that had been diagnosed radiologically outside our Health Board would have been missed, but General Practitioners letters were reviewed to ensure reliability. PD status was ascertained for each patient by the PD specialist and we expect a highly accurate diagnosis of PD. PD severity and duration were recorded to evaluate the impact of PD on fragility fracture since the diagnosis. Sub-analysis was performed to measure the impact of PD severity and duration on adverse outcomes including fragility fracture and mortality. 

Our study also has several limitations. This is a retrospective study based on existing data, and patients were not interviewed or clinically examined. Therefore, we could not study characteristics of the study population, record the cause of death or calculate the case fatality ratio. We did not measure bone mineral density (BMD) or other causes of osteoporosis such as hyperthyroidism, hyperparathyroidism and renal osteodystrophy as part of this study. We also acknowledge other confounding variables including cognition, functional capabilities, sunlight exposure, dietary calcium and vitamin D intake, weight loss and physical exercises, which have not been measured as part of this study. We did not measure the impact of body mass index and drugs, particularly dopaminergic drugs, antidepressants and steroid use on fracture risk, thus adjustments were not possible for sub-analysis. Therefore, we propose that the above limitations be considered before conducting future studies. 

PwP would benefit from multifactorial falls risk assessment, and should be screened for osteoporosis to facilitate early diagnosis and access to evidence-based effective treatment. Osteoporosis risk in PD can be assessed using FRAX^®^ [29] or QFracture^®^ [30] scoring. Integrated PD services with an existing osteoporosis or bone health clinic could help those with severe osteoporosis. We propose quality improvement initiatives, based on the model of improvement (Plan-Do-Study-Act Cycle) to review existing PD services to ensure that all PwP are treated for osteoporosis [31]. We also recommend similar studies to measure the prevalence of osteoporosis in PD in other centres, as we expect that osteoporosis is under reported. In addition, more research to investigate the fracture risk in PwP as compared to healthy controls is required. This will support further studies of interventions or prospective, randomised controlled trials to prevent fragility fractures in this high-risk group.

## 4. Conclusions

There is a high prevalence of undiagnosed osteoporotic fractures in patients attending movement disorder clinics, and 60% do not receive evidence-based medical treatment for the underlying osteoporosis. The prevalence of fragility fracture in PwP is positively correlated to advancing age, female gender, duration and severity of the disease. The best single predictors of a fragility fracture are disease severity and disease duration. The best predictor of mortality is PD severity; followed by age and then fracture history. A further evaluation providing similar services in South Wales is currently being studied. 

## Figures and Tables

**Table 1:** Generalised Linear Model of influence of age, disease duration and severity score on likelihood of fracture. Binomial model with logit link function.

Rank	Variable 1	Variable 2	Variable 3	Degrees of Freedom	Likelihood - Score	*p*
**1**	Age on 20/10/2016	Disease duration (years)	H & Y Score @ Study	3	131.8525	0.000
**2**	Disease duration (years)	H & Y Score @ Study		2	131.3147	0.000
**3**	Age on 20/10/2016	Disease duration (years)		2	98.9471	0.000
**4**	Disease duration (years)			1	98.8608	0.000
**5**	Age on 20/10/2016	H & Y Score @ Study		2	94.5216	0.000
**6**	H & Y Score @ Study			1	94.5209	0.000
**7**	Age on 20/10/2016			1	6.5888	0.010262
